# Nitroglycerine in Chronic Nephritis

**Published:** 1905-06-24

**Authors:** 


### NITROGLYCERINE IN CHRONIC NEPHRITIS.

It is a debatable point how far the high arterial tension associated with chronic interstitial nephritis is a conservative process tending towards compensation for renal inadequacy, but there is no doubt that in many cases increased comfort is obtained for the sufferers from the disease by methods which reduce the excessive tension. For this latter purpose nitroglycerine has for some years past had a great vogue, and not without justification. Unfortunately, as is pointed out by Dr. D. D. Stewart,[1] remarkable tolerance of the drug is readily established, and its use must therefore be most precisely regulated if full benefit is to be derived from its employment. As an instance of this tolerance Dr. Stewart mentions the case of a man who, within six months of a course which began with the administration of 1 minim of a 1 per cent. solution, was taking 50 minims of a 10 per cent. solution four times daily, with less effect upon his circulatory system than had originally been achieved by the initial minute dosage. The author is of opinion that some degree of tolerance must always be looked for, and that the inconvenience of this occurrence can be minimised only by a very careful proportioning of the dose, no more being administered at any time than is barely sufficient to produce some alteration, subjective or objective, upon the arterial tension. The larger the dosage the sooner is tolerance established. In the case in point the patient, knowing the nature of his ailment and immensely impressed both by the name of the drug and by its perceptible activity, advanced the dose beyond the limit of necessity in spite of warnings to the contrary, until he was able to take without distress (and therefore without response) as much as five grains of nitroglycerine four times a day.

Dr. Stewart recommends that the drug be given at frequent intervals, not less than four times a day, and in the smallest quantities which suffice to produce the slightest degree of fulness in the head, or of quickening of the pulse; if this is achieved it may be taken for granted that the desired physiological effects are in process of operation. When the time arrives for the employment of large doses —for the time will inevitably come—it is well to discontinue the administration for a few days at a time at intervals of a week or two, for the tolerance is quickly lost, and on the resumption of the treatment a diminished dose will achieve the purpose.

The 'author observes that nitroglycerine has no effect upon chronic nephritis, except as regards the arterial condition at the moment, and in pointing out that there probably is a conservative element in the increase of blood-tension, observes that "those cases of chronic Bright's disease in which tension is persistently low from the outset are actually of much more gravity than the commonly observed variety with raised vascular tone; and when arterio-capillary resistance shows a tendency to fail voluntarily, scanty urine with more marked albuminuria and dropsy may be expected. In general terms, Dr. Stewart thinks that nitroglycerine is overrated in the treatment of chronic nephritis; he himself has learnt to rely with more confidence upon other methods of reducing arterial tension, in particular the reduction of nitrogenous food, which he considers to be of the highest importance, in conjunction with a free action of the skin and bowels. Blue pill and salts find a place in the therapeutics of many diseases, and deserve as much honour in the case of chronic nephritis as can be claimed for them anywhere.

[1] Jour. Amer. Med. Association, May 27, 1905.

